# Iron and fecundity among Tsimane’ women of Bolivia

**DOI:** 10.1093/emph/eoz020

**Published:** 2019-07-04

**Authors:** Elizabeth M Miller, Maie Khalil

**Affiliations:** Department of Anthropology, University of South Florida, 4202 East Fowler Ave, SOC107, Tampa, FL, USA

**Keywords:** hemoglobin, iron-deficiency anemia, reproductive ecology, maternal health, life history theory

## Abstract

**Background and objectives:**

Iron is critical for women’s reproduction, and iron-deficiency anemia is a global health problem for mothers. While public health programs have aimed to correct iron deficiency in reproductive-aged women with supplementation, a small group of studies have shown that too much iron also has negative effects on birth outcomes. We hypothesize that women’s iron levels evolved within a narrow optimum, and predict that hemoglobin (Hb) levels would be associated with women’s fecundity.

**Methodology:**

We used the publicly available, longitudinal Tsimane’ Amazonian Panel Study to test the association between -Hb levels and hazard of having a next birth (a measure of fecundity) among 116 parous, reproductive-aged Tsimane’ women of Bolivia. A Cox proportional hazards model was used to model Hb level and other predictors against the event of next birth across the observation period, which began at each woman’s previous birth.

**Results:**

The higher the Hb level, the lower the hazard of a woman giving birth within the study observation period (hazard ratio=0.82, *P *= 0.03). However, there was no evidence that low Hb reduced women’s fecundity.

**Conclusions and implications:**

These results demonstrate that high Hb influences women’s fecundity. These results supports the growing body of literature showing that iron metabolism is critical for understanding the evolution of women’s reproduction. More work is needed to determine the evolved optimal range of iron levels for reproductive-aged women.

**Lay summary:**

Lower chance of pregnancy among Tsimane’ women with high Hb levels, suggesting evolved optimal Hb levels in women.

## INTRODUCTION

Iron is essential for life, facilitating oxygen transport throughout the body. On the other hand, too much iron is also toxic, damaging tissues with oxidative stress and feeding pathogenic bacteria within hosts [1, 2]. Organisms have evolved mechanisms to tightly regulate iron homeostasis in the body, absorbing only small percentages of dietary intake and tightly binding iron to protein, rendering it less harmful [3]. Iron deficiency is one of the main causes of anemia, which is defined by not having enough red blood cells to transfer oxygen to tissues [4]. Globally, women with anemia suffer and die due to iron deficiency during pregnancy and birth [5]. 

Iron is a critical resource in women’s reproduction. Mothers transfer large amounts of iron to the fetus during pregnancy, risking iron-deficiency anemia and concomitant perinatal morbidity and mortality [6]. Iron is preferentially transferred to the fetus during pregnancy, even when the mother has severe anemia [7]. Pregnancy-related iron transfer can have cumulative effects on maternal iron storage and metabolism, putting women at risk of maternal iron depletion and anemia [8, 9]. Despite the high need for iron during pregnancy, reproductive-aged women have lower Hb and iron levels than men or postmenopausal women. On the surface, it appears that women’s iron physiology evolved to be at odds with their reproductive needs, prompting nutritional authorities to recommend reproductive-aged women have over twice the intake of dietary iron compared with other adults [10]. This explanation, supported by the medical and nutritional establishment, frames women’s low iron as a pathology that must be corrected during pregnancy [11].

On the other hand, a small but growing body of research suggests that there is, in fact, risk of poor birth outcomes when women’s iron levels, as measured by hemoglobin (Hb), are too high as well as too low [12, 13]. There is, in fact, a U-shaped curve associated with Hb status and birth outcomes such as preterm birth, small for gestational age and stillbirth. The likelihood of these negative effects are higher at both ends of the Hb range, and the lowest risk lies in the center, between 11 and 13 g/dl [12, 14–17]. This finding joins other research that suggests that too much iron can be harmful to the body—oxidative stress, widespread organ damage, teratogenesis, infection and increased infection are all more likely in situations of excess iron [18–22]. Hb, however, is an incomplete indicator of iron in the body. Other indicators of iron storage and metabolism, such as serum ferritin and soluble transferrin receptor, give more detail about the overall levels and availability of iron in the body. Furthermore, certain factors such as infection and inflammation or lack of plasma expansion during pregnancy can alter the relationship between general body iron levels and Hb in red blood cells. However, the majority of the iron in the body is located in Hb, and much of the work on birth outcomes and iron use Hb as a biomarker of interest, making Hb a good starting point for assessing the role of iron in women’s reproductive outcomes [7, 12].

This line of evidence suggests that there is a narrow physiological range in which women’s Hb, as well as overall iron levels, are optimal. If this narrow range provides the best outcomes for maternal and infant mortality, it may also suggest that iron levels could be bound to women’s fecundity, that is, her ability to become pregnant. If so, then women’s iron indicators could be associated with indicators of her reproductive fitness, and could be analysed along with energy stores as predictors of her interbirth interval [7]. We hypothesized that reproductive-aged women’s lower Hb levels is not a pathology but is in fact adaptive, protecting offspring during early pregnancy from the harmful effects of oxidative stress, infection, impaired immune regulation and teratogenesis [19, 23, 24]. This would be reflected as a greater chance of giving birth within a particular time span, a measure that reflects on an individual’s fecundity. Fecundity (the potential reproductive output of an individual) is a related concept to fertility (the actual reproductive output of an individual) and helps form the basis of evolutionary fitness. The purpose of this study is to test the hypothesis that Hb is associated with fecundity, as measured by hazard of experiencing a next birth, in the publicly available Tsimane’ Amazonian Panel Study (TAPS). We predict that high Hb and low Hb will be associated with lower hazard of birth, supporting an adaptive model of iron levels in women. 

## METHODOLOGY

### Population

This study makes use of the publicly available TAPS dataset. The TAPS set consists of longitudinal data from all Tsimane’ living in 13 villages along the Maniqui River in Bolivia from 2002 to 2010 [25]. The main goals of TAPS was to study lifestyle, diets and health of the Tsimane’ people, who are physically active, hunt, fish and forage much of their food, and face challenges related to modernization. The TAPS dataset, while a rich source of information about the Tsimane’, also poses a challenge for independent researchers; notably, that the dataset does not necessarily perfectly match the needs of all research questions. This study sought to overcome this limitation by carefully choosing the subpopulation under study and to pay close attention to variable calculation.

The population under study were reproductive-aged Tsimane’ women, ages 16–45 years, who had given birth to at least one child and who had a Hb measurement in either 2002 or 2003 (Hb was not measured in any other year). They also had to be trackable through the data set through the birth of their next child or 2006–07, whichever came first (the ‘observation period’). Because of these restrictions, the final sample size for analysis was 116 women.

Because this study is based on secondary analysis of a deidentified data set, it does not require approval from an Institutional Review Board.

### Study design

Time between births (birth interval) was a main variable of interest. This presents an analytical challenge because (i) births happen repeatedly over a woman’s lifespan, yet (ii) women may have extended birth intervals, and/or cease reproduction altogether due to menopause or some other reason. Therefore, women might have two to three or more births across the TAPS panel period. This reality dovetails with the nature of the TAPS dataset, which surveys individuals yearly from 2002 to 2010, yet individuals and families do not necessarily appear every year. Hb was measured in the 2002 and/or 2003 measurement years, with some women measured in only one and some measured in both.

Thus, we only considered the birth interval in which women had an Hb measurement. Thus, their birth interval began at the date of their previous birth, covered their Hb measurement, and lasted until their next birth. We also put limits on the observation period rather than leaving it open to 2010, because a majority of women had moved on to their next birth interval after 4 years. For women with Hb measurements in 2002, the end of the study window was the 2006 measurement period, and for women with Hb measurements in 2003, the end of the observation was 2007. Women with Hb measurements in both years had their 2002 measurement used in the analysis. We also restricted the sample to women who were below the age of 45 years, giving them the potential to have one last birth in their reproductive lifespan. However, because not all women would have a completed birth interval, a survival design was chosen for this study. This would allow women who were not censored—did not have a next birth—to be included in analysis.

**Figure 1.**
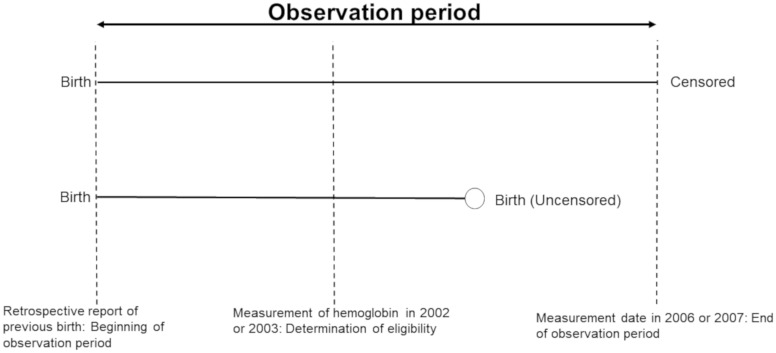
A model of the observation period for uncensored (had a next birth) and censored (no next birth) women constructed from the Tsimane’ Amazonian Panel Study dataset. Although the observation period is aligned in the figure, note that entry into the study varies based on reported date of last birth as well as date of hemoglobin measurement. The relative amount of time between the beginning of the observation period and the hemoglobin measurement date also varied between women. Finally, the end of the observation period varied depending on the date of research follow-up

Birth intervals for eligible women were reconstructed from the TAPS dataset using family variables. This was done primarily by identifying when a new infant, attributed to the eligible woman as her child, appeared in the dataset. The birth dates assigned to the previous birth and next birth were used to reconstruct the birth interval. If women did not have a next birth, the date between their previous birth and the end of the observation period was used. To be included in the sample, women had to be identifiable in the dataset until their next birth had been identified or during their last year in the observation period to confirm they did not have a next birth. Women who had a next birth were considered ‘censored’ for survival analysis, meaning that the event (birth) had occurred. Women who did not have a next birth by the end of the study window were ‘uncensored’, indicating that the event had not occurred. Figure 1 shows the structure of the variables for both censored and uncensored participants. The Hb measurement occurred within the birth/study interval at a different relative time for each woman.

### Variables

Birth interval, with birth as the censoring variable, was the main dependent variable of interest. Hb levels (g/dl) were the main independent variable. For hazard and survival curve analysis, Hb levels were stratified into three roughly equal groups: low (defined as <12 g/dl), medium (≥12 and ≤13 g/dl) and high (>13 g/dl). The low cutoff was chosen as the threshold for anemia, while the cutoff between medium and high reflected the approximate midpoint of the non-anemic women. These cutoffs were determined for descriptive purposes only, and were not used for hypothesis testing.

Multivariate models were also controlled for women’s age, parity, BMI and the past week’s household meat consumption in kilograms. These measures were taken at the same time as the Hb measurement. Previous research in the TAPS dataset used total weekly household food expenditures as an indicator of household income [26]. Unfortunately, that variable was not present in the 2002–03 years, but household food weights and costs were available for many different types of foods. We decided to focus on weekly household meat weight (kg) variables (beef, chicken, pork, duck, fish and game), as this would partially fulfill the concept of income as introduced by Rosinger *et al.* [26] as well as give insight into dietary heme iron available within each household.

One potential confounder that was explored before modeling the data was the timing of Hb measurement within the birth interval. Previous research has shown that in some populations, Hb and other measures of iron status can be lower after birth, and can replete during the postpartum period [8, 9, 27]. Reproductive-related fluctuations within the birth interval thus may confound the results. The potential impact of reproductive-related decrease in Hb was evaluated in the dataset in various ways before proceeding. First, the absolute length of time (in days) between the previous birth and the Hb measurement was calculated, and assessed across the three iron strata. There was a significantly higher interval between previous birth and Hb measurement in the ‘high’ strata, potentially indicating these women may have had a longer time to recover Hb since their previous birth. However, the total interval between births was also significantly longer in the ‘high’ strata, meaning that the other alternative was that the previous birth-Hb measurement interval was an artifact of the longer overall observation period. This artifact made it difficult to include this interval as an independent variable in multivariate models without skewing interpretation, as they are highly collinear. Residuals of the regression between this measurement interval and Hb level indicated no significant evidence of Hb increases across the population within the first 1000 days post-birth. Furthermore, the percentage of the previous birth-Hb measurement interval out of the total observation period was not significantly different between iron strata. Therefore, we decided to model the time between previous birth and the Hb measurement as a percent of the total observation period in multivariate models. This allowed us to control for both alternative explanations in Hb measurement time.

### Statistical methods

Study variables were explored via univariate (means, medians, and standard deviations) and bivariate methods (correlations and *t*-tests) using SAS 9.4. PROC LIFETEST was also used to explore survival and hazard curves for the high-, medium- and low-iron categories. The survival curve estimates the time to next pregnancy for each of the iron strata. Survival curves show the probability of ‘surviving’, in this case, not having a next birth, at a given time point. The hazard curve shows the likelihood of the event (next birth) occurring at each time point (measured in days since previous birth). Survival and hazard curves were used to describe the underlying variation and provide guidance for further hypothesis testing.

Because the descriptive statistics, as well as the strata-based survival and hazard curves, show a linear relationship between birth interval and Hb levels, Hb level was modeled as a linear variable rather than a categorical value for testing our hypothesis. PROC PHREG, the Cox proportional hazard model, was used to test the relationship between Hb levels and time to next birth. Cox proportional hazard models how the hazard of birth at a given time point (hazard rate) changes with Hb level. Time to next birth was modeled as the dependent time variable, and next birth was modeled as the dependent event. Hb level was modeled as a linear independent variable. Parity, age, BMI, weekly household meat usage, Hb measurement year (2002 vs 2003) and Hb measurement interval percentage were included in the model as covariates. Cox proportional hazard models estimate a parameter as well as a hazard ratio for each variable. Hazard ratios for continuous variables can be interpreted as the relative likelihood of the event occurring for each one unit measure of difference in a given time period. For example, a hazard of 2.0 for the Hb variable would indicate that a one-unit increase (e.g. 12.0 vs 13.0 mg/dl) doubles the likelihood of birth at a given time period. A hazard of 0.5 for the Hb variable, on the other hand, halves the likelihood of next birth for each one-unit increase in Hb.

Because Cox proportional hazard models are semi-parametric, they are robust to many of the assumptions needed for linear regression. However, they do require proportional hazards across covariates. To test this, interaction terms between predictors and the log of the time variable were created and included in the PROC PHREG model. The proportionality test option was used to test these interactions. A Wald *χ*^2^ was the test statistic, with the outcome *χ*^2^(7) =5.5, *P *= 0.60. This indicates that the hazards were proportionate and met the assumptions of the model. Multicollinearity of predictors was assessed using variance inflation in PROC REG, to assure that including both age and parity would not impact the performance or interpretation of the model. All variance inflations in the final model were under 3, indicating that the independent variables were not collinear.

Finally, all covariates were tested to see if they confounded the relationship between hazard of birth and Hb. A confounding variable is a variable that influences both the dependent and independent variable in a single model. Confounding exists if the covariate modifies the effect size of the hypothesized relationship by 10% or more. This test was done to assess the possibility of complex relationships between the main hypothesis of interest and model covariates.

## RESULTS

The descriptive statistics in Table 1 show a high rate of anemia (Hb <12.0 g/dl) in the Tsimane’ population (35%), while mean BMI falls within the ‘normal’ category (23.3 kg/m^2^). When dividing the population in to high, medium, and low categories based on Hb level, there are significant differences in interval between births (or between previous birth and end of observation period), with women in the high Hb group having significantly more days between their previous birth and their exit from the analysis, ether via birth or observation period cutoff date. There are marginally significantly fewer women in the high iron group who had a birth between their iron measurement and the study endpoint compared with the medium and low group (63 vs 74 and 80%). Thus, women in the high iron group had longer durations between their births, and were less likely to give birth again during the study period. Women in the high Hb group were marginally significantly older than the medium and low groups, and there appeared to be a trend of increasing mean age from the low group to the high group. There were also marginally significant differences in mean parity between Hb groups, with the medium group having higher mean parity than the high or low groups. There were no significant differences between the three groups on mean weekly household meat amount or BMI.

**Table 1.** Descriptive statistics of Tsimane’ population under study, total and by hemoglobin level

	Total *n* = 116	High (Hb>13) *n* = 40	Medium (13 > Hb>12) *n* = 35	Low (Hb<12) *n* = 41	Test of group differences *F*, *P*
Mean hemoglobin (g/dl)	12.4±1.3	13.8±0.5	12.5±0.26	11.0±0.79	
Percent with Hb>12.0	35%	–	–	–	–
Age (years)	28.3 ± 7.6	30.1 ± 7.2	28.3 ± 8.0	26.5 ± 7.6	2.32, 0.10
Parity at end of study period	5.5 ± 2.9	5.3 ± 2.4	5.9 ± 3.4	5.4 ± 2.9	2.88, 0.061
BMI (kg/m^2^)	23.3 ± 2.7	23.4 ± 3.2	23.0 ± 2.0	23.5 ± 2.6	0.34, 0.71
Percent becoming pregnant during study period (censored)	72%	63%	74%	80%	*Z* = 1.8, 0.071
Mean interval between births or prior birth and end of study period (days)	1463 ± 881	1796 ± 1053	1303 ± 748	1273 ± 711	4.67, 0.011
Mean interval between prior birth and Hb measurement (days)	651 ± 730	892 ± 944	521 ± 580	527 ± 535	3.47, 0.035
Interval between prior birth and Hb measurement as percentage of total interval between births (%)	43%	44%	40%	43%	0.54, 0.58
Household meat/week (kg)	13.6 ± 13.3	12.2 ± 11.1	14.6 ± 12.2	14.1 ± 16.0	0.36, 0.70

Means and standard deviations are reported for continuous variables, while percentages are reported for the dichotomous variable. One-way ANOVA statistics represent significant differences between the three hemoglobin levels for continuous variables; for dichotomous variables Cochran–Armitage test for trend (*Z*), a modified *χ*^2^ test for ordinal variables was used.

**Figure 2.**
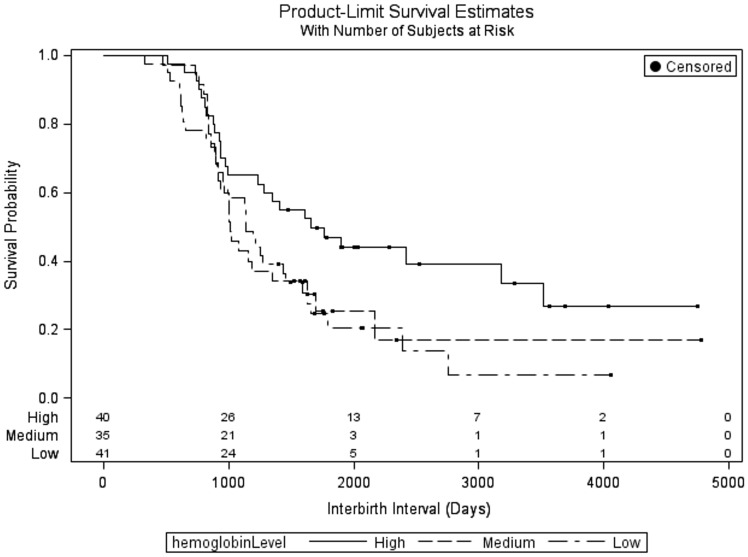
Survival plot of time to birth (in days) by high-, medium- and low-hemoglobin strata

**Figure 3.**
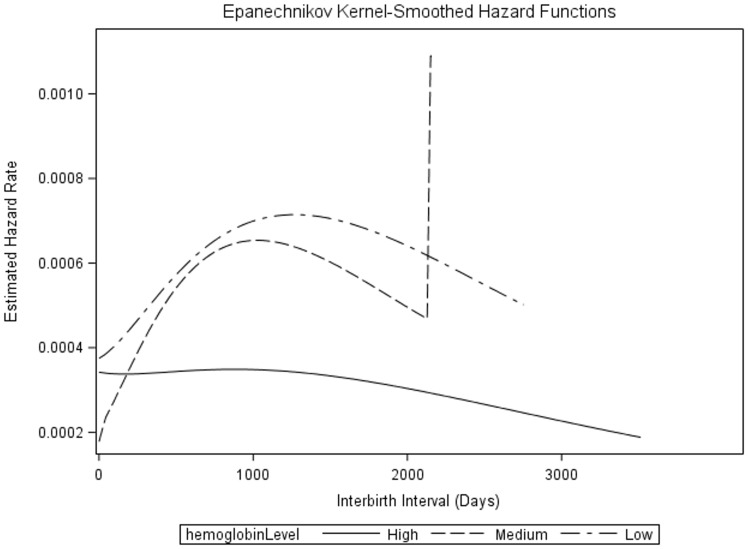
Plot of hazard rate (daily risk of birth) by high-, medium- and low-hemoglobin strata. The vertical line in the medium strata is an artifact of one participant in that group with a long observation time

The survival curve (Fig. 2) for the three iron strata shows that the high iron stratus begins to diverge from the survival curves of the other two strata at around 1000 days, with a lower rate of giving birth after that time. The median survival time, or the point at which half of the group has given birth, for each of the three strata are 1660 days (high), 1010 days (medium) and 1140 days (low). Figure 3 shows the hazard curve for the three strata. This curve represents the hazard (risk) of the next birth at a given point in time over the study period. While the hazard curves of the low and medium strata have relatively similar shapes and peaks (the sharp upturn in the medium strata is due to one individual), with a sharply increasing hazard of birth from previous birth to just after the 1000 day mark and a decline thereafter, the hazard curve of the high iron stratus begins at a similar hazard as the other two strata but declines across the observation period.

The Cox proportional hazards multivariate model (Table 2) shows a significant linear relationship between Hb level and hazard of birth, with women who have lower Hb having greater hazard of birth than women with high Hb. Older women had a significantly lower hazard of birth than younger women while higher parity women had a significantly greater hazard of birth than women with lower parity. BMI, household meat intake per week and year of Hb collection were not significantly associated with hazard of birth.

**Table 2.** Cox proportional hazards model for survival of time between births among non-pregnant, parous, reproductive-aged Tsimane’ women

	Estimate (*P*-value)	Hazard ratio
Hemoglobin level (g/dl)	−0.20 (0.03)	0.82
Age (years)	−0.046 (0.05)	0.96
Parity	0.20 (<0.0001)	1.22
BMI	0.014 (0.74)	1.01
Household meat/week (kg)	−0.010 (0.27)	0.99
Prior birth-Hb measurement interval percentage	0.0051 (0.24)	1.01
Year of Hb measurement (2002 vs 2003)	0.45 (0.08)	1.57

A hazard ratio below 1 indicates a lower hazard of birth at a given time point for each one-unit increase in the independent variable, while a hazard ratio above 1 indicates a greater hazard of giving birth for each one-unit increase in the independent variable.

To assess confounding, models were run with and without each covariate to assess the degree of change in the effect size between Hb and hazard of birth. The addition of age changed the effect size from −0.24 to −0.17 (41% change), increasing the hazard ratio and making the relationship between Hb and hazard of birth less statistically significant. Similar results were found when parity was assessed as a confounder (−0.28 to −0.17, a 64% change in effect size). No similar effect of confounding was found for any other variable. While the addition of parity and age made the effect size of the main relationship less significant, it lends assurance that age and parity were adequately controlled for in the model and reiterates the statistically significant main effect of Hb on hazard of birth.

## DISCUSSION

Women with higher Hb levels had a lower hazard of giving birth at a given time point, supporting the hypothesis that too much iron is a detriment to women’s fecundity. However, women with lower Hb levels did not have a lower hazard of birth at a given time point, contradicting the hypothesis that low Hb also lowers women’s fecundity. There are several potential explanations for this finding. First, there is a relatively small number of women with severe anemia in this sample. In fact, only two women had Hb values below 9.0 g/dl, and no woman had a Hb value below 7.0 g/dl. One interpretation is that mild anemia does not have an effect on fecundity, but severe anemia does. This question may need to be evaluated in a population with a higher prevalence of severe anemia. The women with the highest Hb levels were also somewhat older and have slightly, but not significantly, less weekly household meat purchased than women with lower Hb. There is a known relationship between increasing age and Hb in women, even among pre-menopausal women [27], but it is surprising that women with fewer heme sources of iron in the household would have higher Hb. However, these variables were statistically accounted for in the model, and thus the explanation for the finding lies elsewhere. Other potential covariates, such as inflammation, could not be directly evaluated using this dataset, although both chronic and acute inflammation can impact Hb [28, 29].

There is a considerable body of evolutionary literature that evaluates the energetic context of fecundity in relation to interbirth interval length. This literature is built around life history theory, a framework that hypothesizes women have limited energy available for reproduction and somatic maintenance, and must allocate it effectively to optimize fitness across their reproductive lifespans [30]. For women in resource-poor environments, this can mean a longer interbirth interval, as they spend more time recovering energy stores after pregnancy (also known as the ‘metabolic load hypothesis’ for lactational amenorrhea) [31]. Some women do not completely recover energetic reserves between pregnancies [32]. Iron, although not an energetic resource in life history terms, appears to have at least some of these characteristics. It is a limited resource, is required for both reproduction and somatic maintenance, and is depleted and recovered (sometimes incompletely) during pregnancy and postpartum [7]. The current project provides some evidence that certain Hb levels are necessary for reproduction, and may even have some predictive power when assessing interbirth interval. Among the Tsimane’, the hazard curves appear to provide some insight into the fecundity of the population. Among women with Hb levels below 13.0 g/dl, the hazard curves show a sharp increase in the hazard of giving birth after a previous birth, peaking around 1000–1100 days and declining thereafter. Of note, this range is within the WHO’s recommendations for birth spacing, with a recommendation of at least 18 months after birth to start a new pregnancy [33]. The women with Hb levels over 13.0 g/dl, on the other hand, have a flatter hazard curve, with a lower hazard of pregnancy over the entire birth interval. In essence, it appears that high iron modifies population-specific patterns of fecundity so that women with high iron have lower fecundity. This may be a protection against becoming pregnant when iron levels are too high, protecting the embryo from the negative effects of iron. In contrast, lower Hb does not appear to modify population-specific patterns of fecundity. There could be two potential reasons for this. First, it may be that lower iron is not the same evolutionary impediment to fecundity as higher iron, with low iron primarily impacting maternal health via depletion, and high iron impacting embryonic and fetal survival via inflammation, teratogenesis or oxidative stress. If the fitness loss from fetal mortality is greater than fitness loss from maternal poor health, subfecundity and/or mortality, then natural selection may operate for lower iron in women. The second explanation is that Tsimane’ women did not have low enough Hb to understand the potential for fecundity issues associated with severe anemia. This explanation would favor the role of stabilizing selection in selecting for an optimum Hb. Because maternal mortality due to iron deficiency anemia is quite high, the second explanation is more likely; however, these possibilities remain to be tested directly in humans.

If women’s evolved iron optimum exists within a relatively narrow window, what is the proximate mechanism that keeps them there? Women have lower iron stores and lower Hb than men, a pattern that emerges during puberty and persists until menopause. Higher testosterone levels are implicated in the formation of red blood cells, which has been used to explain some of this dimorphism [34]. Blood loss due to menstruation is another possible explanation, but there is some debate about the amount of iron that is actually lost in menstrual fluid [7, 35, 36]. Nausea, a common side effect of excessive iron consumption, has also been postulated as an evolved mechanism to keep individuals from consuming too much iron, particularly during pregnancy [37]. Finally, there is a growing but inconclusive body of literature that demonstrates that estrogen is implicated in the regulation of iron homeostasis, and could be another factor that regulates reproductive-aged women’s iron levels to function within a certain range [38–40]. Assessing the relative contribution of these proximate mechanisms of iron control in humans—menstrual blood loss, iron-related nausea, and estrogen—is not an easy task. Overt menstruation is a derived characteristic within the Primate order [41], while the role of estrogen in internal gestation is a primitive characteristic among placental mammals [42]. Similarly, veterinary sources suggest that nausea and vomiting from iron supplementation is common among placental mammals [43]. A more complete survey of an iron optimum across Mammalia would be necessary to test the relative evolutionary importance of these three factors in humans.

If there is proximate management of iron levels via physiology and ultimate evidence for optimal Hb via fecundity, why are so many women at risk for iron-deficiency anemia-related maternal morbidity and mortality? The answer most likely lies with the interaction between biology and society. Rather than assume any Hb level above the anemic range is ‘correct’, we should look to the structural factors that push women’s iron physiology outside of ranges that are optimal for fecundity. For high iron, some of these causes can be genetic, such as hereditary hemochromatosis and other genetic blood disorders, while other factors may be somewhat more modifiable, such as insulin resistance and the metabolic syndrome, alcohol consumption and excessive iron supplementation [44, 45]. For some of these factors, such as metabolic syndrome and insulin resistance, the causal factors are bidirectional, meaning that excessive supplementation can increase risk of chronic illness [46, 47]. On the other hand, lack of secure food access, environments that promote excessive helminth infection and intestinal bleeding, interbirth intervals that are too short, and lack of access to reproductive health care all contribute to high rates of maternal anemia globally [5, 48–50]. Excessive supplementation should not be seen as an obvious solution to the problem, as this can potentially lead to Hb and/or iron levels that are too high for women to safely conceive or have a healthy pregnancy. Improvements to women’s iron status should focus on improving women’s circumstances, ensuring they have access to a secure, healthy diet, clean water and sanitation, and have access to appropriate health care. In the case of the study population, it appears that most Tsimane’ women do not appear to have Hb values that fall into the severely anemic range, despite high rates of helminth infections in the population [51]. On the other hand, the causes of higher Hb in this population should be investigated more thoroughly.

Parity was related to Hb levels, with women who had intermediate Hb levels having higher parity than women who were either high or low. While age-specific parity does not necessarily reflect lifetime fertility, this is some evidence that optimal Hb levels impact not only fecundity, but also fertility. Parity itself was also a significant predictor of birth hazard. Tsimane’ women with greater parity have a higher hazard of birth compared with those with lower parity. The opposite pattern appears to hold true for age—that older aged women have a lower hazard of birth compared with younger women—but this may be a statistical artifact after controlling for parity. This is an interesting finding, and is consistent with previous findings that Tsimane’ have high fertility rates [52]. This is a finding that deserves more investigation, particularly in light of the relationship between maternal condition due to socioeconomic development and parity among the Tsimane’ [53].

There are several limitations to this study, largely due to the use of an existing dataset. One limitation is that it was difficult to control the timing of Hb collection within women’s interbirth interval. If women were closer to birth on either end of their birth interval, their Hb may have been lower. The women with shorter birth intervals, by definition, would be closer to either birth. There is no easy way around this limitation in this dataset, especially since statistical control leads to multicollinearity in models. Similarly, there are no data that address the possibility of infant mortality after birth; it is possible that women who lost a baby returned to fecundity more quickly without a concomitant return to pre-pregnancy Hb. This may bias results to a higher early hazard of pregnancy in women with lower Hb. This may also be a problem with preterm births; if preterm birth is more likely among women with lower Hb, then it may bias results towards shorter birth intervals. It is also impossible to correct for known modifiers of Hb levels, such as inflammation, menstrual periods, use of iron supplements or hormonal birth control, and parasite infection. Dietary intake of iron was similarly difficult to assess, as measures of food availability exist only at the household level. Also, without additional measures of iron homeostasis, it is difficult to assess whether Hb levels are due to iron deficiency (or overload), or some other cause. Furthermore, the age range of women in this study captures all reproductive-aged women; a larger study that could tolerate subgroup analysis would be more effective at teasing apart the complex relationship between Hb, age, and parity. Also, these results may not generalize to many populations of women; the Tsimane’ in this population were not severely anemic, which could limit the applicability of this work for populations where women have a greater risk of severe anemia.

Finally, there is another potential interpretation that could be derived from this data: that high Hb women are pursuing a slower life history strategy, with higher somatic investment and time between births. We believe this explanation is less likely based on (i) the relationship between high Hb and poorer birth outcomes [12] and (ii) the overall health impact of high iron in clinical and infectious disease settings [20, 54]. However, the current data cannot distinguish between the two explanations. Demographic measures such as infant mortality or completed fertility would be useful in future research to distinguish between the two options. 

## CONCLUSIONS AND IMPLICATIONS

These results contribute to the growing body of literature suggesting that women’s iron levels evolved to fit an optimal range. More broadly, life history theory may benefit from considering iron as a resource that can be allocated across the lifespan to support reproduction. The knowledge that high iron may impact fecundity also has implications for public health policy for reproductive-aged women. 
